# PP22 New Payment Models For Innovative Technologies Embedded In An Integrated Care Context: A Delphi Study

**DOI:** 10.1017/S0266462325102420

**Published:** 2025-12-29

**Authors:** Paula Closa, Marcelo Soto, Laura Sampietro-Colom

## Abstract

**Introduction:**

The HI-PRIX European project aims to accelerate access to high value, affordable health innovations. This Delphi exercise aimed to build a consensus on new payment schemes that could facilitate the adoption or uptake of innovative medical devices, including digital health technologies, in an integrated care (IC) context.

**Methods:**

A two-round Delphi exercise was performed, which included 29 statements distributed into four sections: (i) principles for innovation to qualify for a new payment scheme; (ii) payment schemes to support the adoption of innovative technologies by healthcare providers in an IC context; (iii) additional payment schemes for digital health technologies; and (iv) targeted funding sources. The Delphi was sent to 180 thematic-knowledgeable professionals. A six-point Likert scale was used to quantify agreement and consensus rules were established. Round two focused only on statements with moderate consensus.

**Results:**

Round one received 92 responses, with around 65 percent of the statements achieving moderate consensus, 28 percent high consensus, and seven percent low consensus. High consensus statements emphasized the importance of incorporating health technology assessment processes to inform pricing negotiations, establishing clear criteria for qualifying innovations into an innovative payment scheme, and incorporating performance-based payments to incentivize their adoption. The findings also highlighted the need to align payment schemes with broader healthcare objectives, such as improving patient pathways and ensuring equitable access, while addressing implementation barriers. Participants stressed the importance of tailoring payment schemes to specific healthcare contexts and ensuring financial sustainability.

**Conclusions:**

The Delphi exercise highlighted both the need and the feasibility of implementing new payment schemes to support the adoption of innovative technologies, specifically in IC contexts. Combining different payment schemes is advisable to leverage the benefits of innovative technologies, while minimizing potential perverse incentives and ensuring alignment with broader healthcare objectives.

